# Inferior Occipital Gyrus Is Organized along Common Gradients of Spatial and Face-Part Selectivity

**DOI:** 10.1523/JNEUROSCI.2415-20.2021

**Published:** 2021-06-23

**Authors:** Benjamin de Haas, Martin I. Sereno, D. Samuel Schwarzkopf

**Affiliations:** ^1^Department of Psychology, Justus Liebig Universität, 35394 Giessen, Germany; ^2^Experimental Psychology, University College London, London WC1E 6BT, United Kingdom; ^3^SDSU Imaging Center, San Diego State University, San Diego, California 92182; ^4^School of Optometry and Vision Science, University of Auckland, Auckland 1142, New Zealand

**Keywords:** face processing, inferior occipital gyrus, retinotopy, ventral stream

## Abstract

The ventral visual stream of the human brain is subdivided into patches with categorical stimulus preferences, like faces or scenes. However, the functional organization within these areas is less clear. Here, we used functional magnetic resonance imaging and vertex-wise tuning models to independently probe spatial and face-part preferences in the inferior occipital gyrus (IOG) of healthy adult males and females. The majority of responses were well explained by Gaussian population tuning curves for both retinotopic location and the preferred relative position within a face. Parameter maps revealed a common gradient of spatial and face-part selectivity, with the width of tuning curves drastically increasing from posterior to anterior IOG. Tuning peaks clustered more idiosyncratically but were also correlated across maps of visual and face space. Preferences for the upper visual field went along with significantly increased coverage of the upper half of the face, matching recently discovered biases in human perception. Our findings reveal a broad range of neural face-part selectivity in IOG, ranging from narrow to “holistic.” IOG is functionally organized along this gradient, which in turn is correlated with retinotopy.

**SIGNIFICANCE STATEMENT** Brain imaging has revealed a lot about the large-scale organization of the human brain and visual system. For example, occipital cortex contains map-like representations of the visual field, while neurons in ventral areas cluster into patches with categorical preferences, like faces or scenes. Much less is known about the functional organization within these areas. Here, we focused on a well established face-preferring area—the inferior occipital gyrus (IOG). A novel neuroimaging paradigm allowed us to map the retinotopic and face-part tuning of many recording sites in IOG independently. We found a steep posterior–anterior gradient of decreasing face-part selectivity, which correlated with retinotopy. This suggests the functional role of ventral areas is not uniform and may follow retinotopic “protomaps.”

## Introduction

A major objective of neuroscience is to clarify the functional organization of visual cortex. Early and dorsal visual areas are organized as retinotopic maps (Inouye, 1909; Holmes, 1918; Sereno et al., 1995; Wandell et al., 2007). Later, occipitotemporal areas are identified as patches with categorical stimulus preferences (Grill-Spector and Weiner, 2014), the most prominent example of which is a succession of face patches (Kanwisher et al., 1997; Tsao et al., 2008; Grill-Spector et al., 2017). However, the functional organization within these ventral areas is less well understood. Recent neuroimaging results in humans suggest that some dorsal and superior areas are organized along abstract gradients of numerosity (Harvey et al., 2013; Harvey and Dumoulin, 2017) and event duration (Protopapa et al., 2019; Harvey et al., 2020). But as of yet, no such intra-areal, nonretinotopic axes of organization have been reported for the ventral stream.

The coarse organization of the ventral stream into patches preferring faces (Kanwisher et al., 1997; Tsao et al., 2008; Grill-Spector et al., 2017), places (Aguirre et al., 1998; Epstein and Kanwisher, 1998); text (McCandliss et al., 2003; Saygin et al., 2016), body parts (Orlov et al., 2010; Weiner and Grill-Spector, 2013), and objects (Grill-Spector et al., 2001) of small and big real-world size (Konkle and Oliva, 2012; Long et al., 2018) coincides with matching retinotopic biases, or even maps (Levy et al., 2001; Hasson et al., 2002; Arcaro et al., 2009; Kolster et al., 2010; Konkle and Oliva, 2012; Huang and Sereno, 2013; Grill-Spector and Weiner, 2014; Silson et al., 2015, 2016; Sood and Sereno, 2016; Livingstone et al., 2019). For instance, face-preferring areas have a stronger central bias than place- or scene-preferring areas (Hasson et al., 2002). Such retinotopic biases have inspired a “protomap” hypothesis of ventral stream organization, according to which categorical preferences emerge from initial retinotopy and simple wiring rules, interacting with visual experience and gaze behavior (Arcaro and Livingstone, 2017; Arcaro et al., 2019; Gomez et al., 2019; Livingstone et al., 2019).

A number of recent studies have shown that human perception and neural processing are tuned to typical stimulus locations and spatial arrangements (Chan et al., 2010; Kaiser et al., 2014, 2019; Kaiser and Cichy, 2018). For instance, human observers are better at recognizing eyes in the upper visual field (UVF) and mouths in the matching lower visual field (LVF) locations for typical gaze behavior (de Haas et al., 2016; de Haas and Schwarzkopf, 2018). Furthermore, the neural representation of face parts in the inferior occipital gyrus (IOG) is more distinct for features presented at typical visual field locations (de Haas et al., 2016) and further apart within the face (Henriksson et al., 2015). This has led to an extension of the protomap hypothesis to the functional organization within areas such as IOG. IOG may start out as a retinotopic protomap for the central visual field and acquire matching “faciotopy” via the input biases implied by typical human gaze behavior. However, this hypothesis has only been tested indirectly, using decoding methods (de Haas et al., 2016) or coarse winner-take-all approaches (Orlov et al., 2010; Henriksson et al., 2015).

Here, we fitted Gaussian population tuning curves (pTCs) to estimate the tuning properties of neuronal populations in IOG, separately for each point of a 3D mesh model of the cortical surface (henceforth, called “vertex”). Vertex-wise pTCs have first been used to map retinotopy in early visual areas (Dumoulin and Wandell, 2008), but they also allow tests of functional organization along other, more abstract stimulus dimensions (Harvey et al., 2013, 2015; Harvey and Dumoulin, 2017). Here, we probed vertex-wise tuning for retinotopic locations and critically also for face parts. Our results confirmed Gaussian tuning for retinotopic locations and for the preferred relative position in the face for most vertices in IOG. Crucially, and in line with the protomap hypothesis, both types of tuning were significantly correlated with each other. In most hemispheres, spatial and face-part selectivity in IOG decreased along a posterior-to-anterior gradient.

## Materials and Methods

### 

#### 

##### Participants.

Seven healthy participants with normal or corrected-to-normal visual acuity completed the experiment (mean age, 31 years; SD, 5 years; three females; all right handed), including two authors (B.d.H. and D.S.S.). Participants were recruited from the local participant pool and gave written informed consent to take part in the study, which was approved by the University College London ethics committee.

##### Stimuli.

Participants viewed stimuli via a mirror mounted at the head coil at a viewing distance of ∼68 cm and at a resolution of 1920 × 1080 pixels [∼30.3° × 16.9° visual angle (d.v.a.)]. All stimuli were programmed and presented in MATLAB (MathWorks) using the Psychophysics Toolbox 3 extension (Brainard, 1997; Pelli, 1997; http://psychtoolbox.org) and a projector with a refresh rate of 60 Hz.

Stimuli consisted of horizontal bars showing face parts and were shown on a mid-gray background. Each bar had a width of 10 d.v.a. and a height of 1.1 d.v.a. Face-part images filling this aperture were sampled from portraits in the SiblingsDB set (Vieira et al., 2014), which were cropped to show an area covering chin to hairline, scaled to gray, and an equal size of 10 × 10 d.v.a.

For spatial mapping, a single face part traversed up and down the visual field (eyes or mouth, alternating between runs and rapidly flickering through different facial identities at a frequency of 6 Hz (each identity ON for 5 frames followed by 5 OFF frames; compare Movie 1). During each run, the bar started at 5 d.v.a. above a fixation dot and slowly moved downward with a step size of 0.55 d.v.a. every second TR (repetition time; 1 s) until it reached 5 d.v.a. below fixation. This “down” trial lasted 32 s and was followed by “up,” “blank,” up, down, and blank trials of equal duration, with blank trials showing only the central fixation dot, which had a diameter of 0.08 d.v.a. During up and down trials, the fixation dot was superimposed whenever mapping stimuli traversed it.

Movie 1. Example sweep of retinotopic mapping stimulus. The retinotopic stimulus traversed up and down the vertical meridian, rapidly cycling through facial identities (6 Hz) and showed either eye or mouth regions (alternating between runs). The example shows a downward sweep from a run with eye region stimuli. Fixation compliance was monitored online using an eyetracker and fed back in a gaze-contingent fashion. 10.1523/JNEUROSCI.2415-20.2021.video.1

For face-part mapping, different face parts (corresponding to approximately one-ninth of the full-face image) were centered at a single visual field location (∼0.8 d.v.a. above or ∼1.9 d.v.a. below fixation, alternating between runs and approximately corresponding to eye and mouth positions for the full face images, assuming a typical fixation location just below the eyes (Peterson and Eckstein, 2012). Face parts were rapidly sampled from different facial identities at a frequency of 6 Hz. Additionally, the sampling location within the face images slowly traversed up and down in steps of approximately one-eighteenth of the full-face images every second TR (compare Movie 2). Each run started with a down trial, beginning at the hairline and moving the sampling window to the chin within a duration of 32 s, followed by up, blank, up, down, and blank trials of equal duration.

Movie 2. Example sweep of face part-mapping stimulus. The face part-mapping stimuli traversed up and down a vertical face space, spanning from chin to hairline, while rapidly cycling through facial identities (6 Hz), and were presented slightly above or below fixation (alternating between runs). The example shows a downward sweep from a run, with stimuli shown slightly above fixation. Fixation compliance was monitored online using an eyetracker and fed back in a gaze-contingent fashion. 10.1523/JNEUROSCI.2415-20.2021.video.2

To determine the position of IOG relative to visual field maps in early visual cortex, we ran an additional retinotopic mapping experiment in one of our participants, using population receptive field (pRF) mapping. Details of the stimulus, paradigm, and fitting procedure for the retinotopy experiment closely followed the “natural images” condition in the study by van Dijk et al. (2016) and can be retrieved from there. Briefly, observer S1 completed six runs of a retinotopic experiment, viewing natural image carriers in a combined wedge and ring aperture, which was displayed on a gray background and extended up to a maximum eccentricity of ∼14 d.v.a. Natural images changed at a fast pace of ∼8.6 Hz. The radius of the ring and the width of the wedge scaled logarithmically with eccentricity. During the run, the wedge aperture completed three clockwise revolutions, while the ring contracted four times, followed by three anticlockwise revolutions and expansions. Wedge revolutions advanced in steps of 15° per volume (TR = 1.5 s), and the inner width of the ring varied between ∼0.3 and ∼12.5 d.v.a. in 18 logarithmic steps, advancing one step per volume. This stimulus was preceded and followed by a blank display lasting four and eight volumes, respectively. Throughout the run, the observer completed a central fixation task, reading a text that was presented as a succession of single letters, each presented for 400 ms (Arial, 9-point type).

##### Procedure.

Before each run, participants were shown the full image of one of two target faces. Participants were asked to press a button whenever they recognized a part of this face within the flickering stream of facial identities in the stimulus aperture. This task was meant to ensure stimulus-directed attention, and all participants reported it to be demanding. Behavioral responses were not recorded because of a hardware failure.

Each participant was asked to keep fixation on a central blue dot (radius, ∼0.08 d.v.a.) during each run. Fixation compliance was monitored and fed back online using an EyeLink 1000 MRI-compatible eyetracker (http://www.sr-research.com/), tracking the gaze position of the left eye. If participants deviated from central fixation by more than a critical threshold (0.5–2 d.v.a., individually determined based on calibration accuracy), the color of the fixation dot turned red, reminding participants to fixate. Each participant was well experienced in psychophysical and/or MRI procedures, and overall fixation compliance was excellent.

Each participant was scanned multiple times, and completed a total of 12–24 face part-mapping runs and an equal number of spatial-mapping runs (average of 17 runs per condition per participant).

##### Image acquisition and preprocessing.

Functional and anatomical scans were acquired using a 1.5 T MRI scanner (Avanto, Siemens) with a 32-channel head coil (Siemens Medical Systems). The front attachment of the head coil was removed to avoid restrictions of the field of view, leaving 20 channels. Functional T2-weighted multiband 2D echoplanar images were taken with a multiband (Breuer et al., 2005) sequence (TR = 1 s, TE = 55 ms, flip angle = 75°, acceleration = 4, matrix size = 96 × 96, 36 transverse slices covering occipital and temporal cortices) with a resolution of 2.3 mm isotropic voxels. We obtained 202 volumes per run, including 10 dummy volumes at the beginning. A T1-weighted anatomical MPRAGE (magnetization-prepared rapid acquisition gradient echo) image was acquired (1 mm isotropic voxels, flip angle = 8°, TE = 3.57 ms, TR = 8.4 ms, TI = 1000 ms, inversion spacing = 2730 ms, 176 partitions) with a resolution of 1 mm isotropic voxels.

All image files were converted to NIfTI format and preprocessed using SPM 12 (http://www.fil.ion.ucl.ac.uk/spm/software/spm12/) and custom MATLAB code. The first 10 volumes for each run were discarded to allow for the T1 signal to reach steady state. The remaining functional images were mean bias corrected, realigned, unwarped, and coregistered to the anatomical scan. Time series were bandpass filtered with a discrete cosine transform, removing the first two components to remove slow drifts and high-frequency noise >0.26 Hz, *z*-scored, and averaged across spatial and face part-mapping runs, respectively. The anatomical scan was used to reconstruct the cortical surface using FreeSurfer (http://surfer.nmr.mgh.harvard.edu), and the functional time series were projected onto the surface (median position between pial and gray matter/white matter boundary for each vertex).

Functional scans for the retinotopy run were acquired using a 3 T MRI scanner (Prisma, Siemens) with a 64-channel head coil (Siemens). Functional T2-weighted 2D echoplanar images were taken with a multiband (Breuer et al., 2005) sequence (TR = 1.5 s, TE = 30 ms, flip angle = 71°, acceleration = 2, matrix size 100 × 100, 46 transverse slices covering occipital and temporal cortex) with a resolution of 2.2 mm isotropic voxels. We obtained 156 volumes per run. Images were converted to NIfTI format and preprocessed using SPM 12 and custom MATLAB code (mean bias correction, realignment, unwarping, and coregistration to the anatomical scan). The functional time series were then projected onto the surface (median position between pial and gray matter/white matter boundary for each vertex).

##### Model fitting.

Spatial and face-part tuning models were estimated from the fMRI data using SamSrf 5.84 (https://figshare.com/articles/SamSrf_toolbox_for_pRF_mapping/1344765) and custom MATLAB code. The basic principle involved the generation of model-based neural response predictions for the stimulus time course, the convolution of this neural prediction with a hemodynamic response function (HRF), and a comparison of the resulting response predictions with the empirical time course to determine best fitting parameters (those explaining the largest fraction of variance, *R*^2^). These methods are similar to those we previously used to model population-receptive fields in early human visual cortex and described in detail previously (de Haas et al., 2014).

Population tuning curves describe the aggregate response profile of the neuronal population at a given cortical location (voxel or vertex). We fitted a Gaussian tuning curve model with the following two parameters of interest: its peak preference along the stimulus dimension [center location (μ)] and its width [SD (σ)], indicating the selectivity of the population response along the stimulus dimension. For spatial mapping, the stimulus dimension was the vertical meridian; for face-part mapping, it was the relative vertical position within the face (ranging from chin to hairline; compare Stimuli subsection). Model fitting followed a two-pass procedure (Harvey et al., 2015). The first pass determined the best fitting model among a grid of predictions based on Gaussians models, with μ ranging from −0.2 to 1.2 in steps of 0.005 within the stimulus space (0 and 1 corresponding to the stimulus edges; i.e., 5 d.v.a. above and below fixation for spatial mapping and to the hairline and chin for face-part mapping); and the search space for σ defined by 2×, with the exponent *x* ranging from −5.6 to 1 in steps of 0.05 within the stimulus space. In this first pass, neural predictions were convolved with a canonical HRF.

Following this first fit, we determined individual parameters of the (double-γ) HRF. For HRF fitting, we pooled empirical time courses and fitted neural predictions for a given participant across hemispheres and conditions. Fixed neural predictions were then convolved with an array of double-γ functions (coarse fit grid search), followed by a gradient descent optimization of HRF parameters (fine fit; Nelder and Mead, 1965). The optimization criterion in both cases was the average *R*^2^ value across pooled vertices that crossed an inclusion threshold of *R*^2^ > 0.2 in the first pass (Harvey et al., 2015).

The second pass refitted the parameters of the neural model, but now using the individual HRF for hemodynamic convolution of the predicted response.

**Figure 2.**
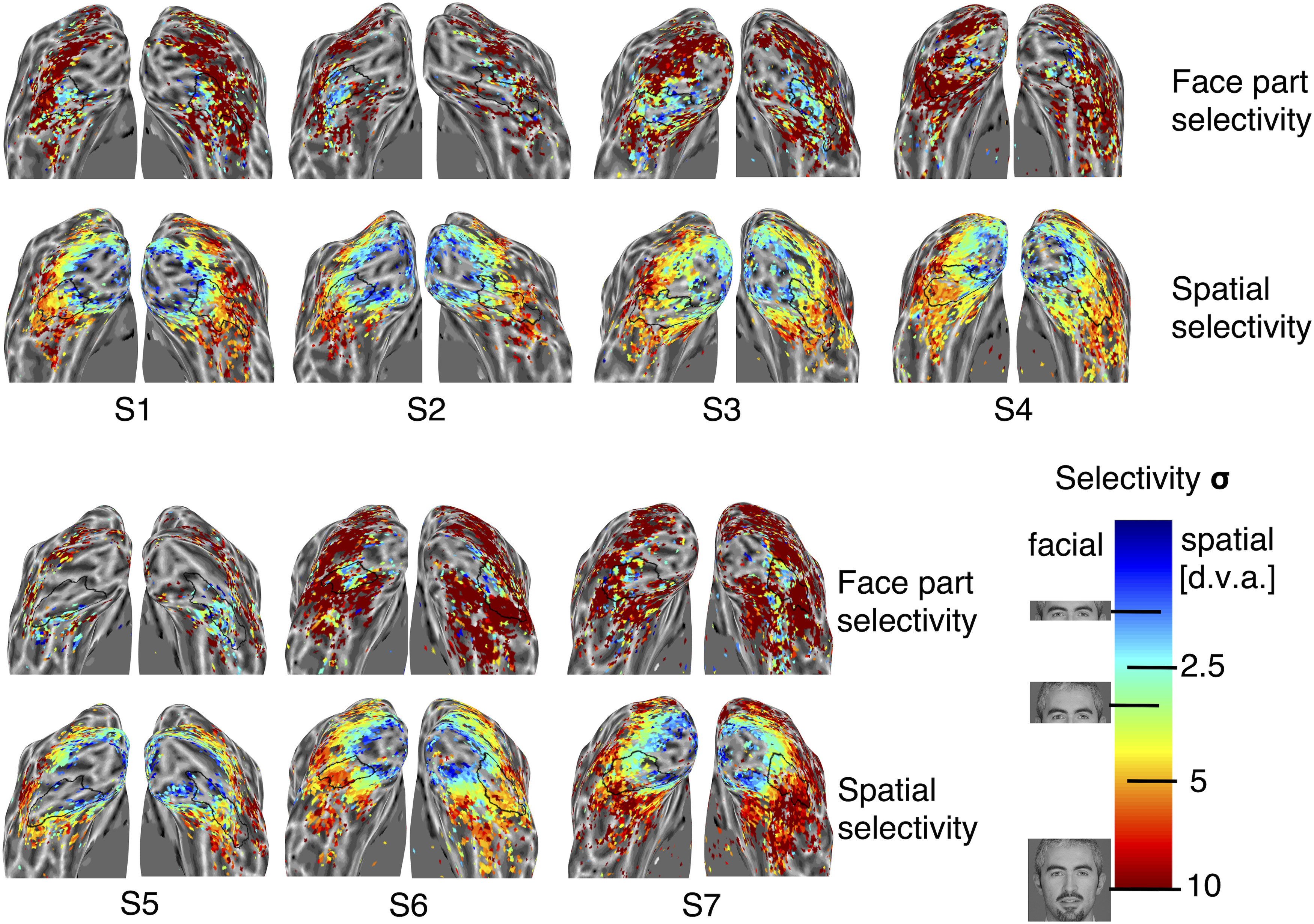
Unsmoothed maps of face-part and spatial selectivity for all hemispheres. The color of each vertex indicates the degree of face-part and spatial selectivity (upper and lower maps, respectively, as indicated by the labels). Colors correspond to the width parameter (σ) of the best fitting Gaussian tuning curve model, as indicated by the color bar (face images show examples of the face extent covered by σ for corresponding colors in face-part selectivity maps; d.v.a. values show corresponding degrees of visual angle for σ in spatial selectivity maps). Black outlines demarcate the boundaries of IOG, determined by the FreeSurfer algorithm based on individual anatomy.

**Figure 3.**
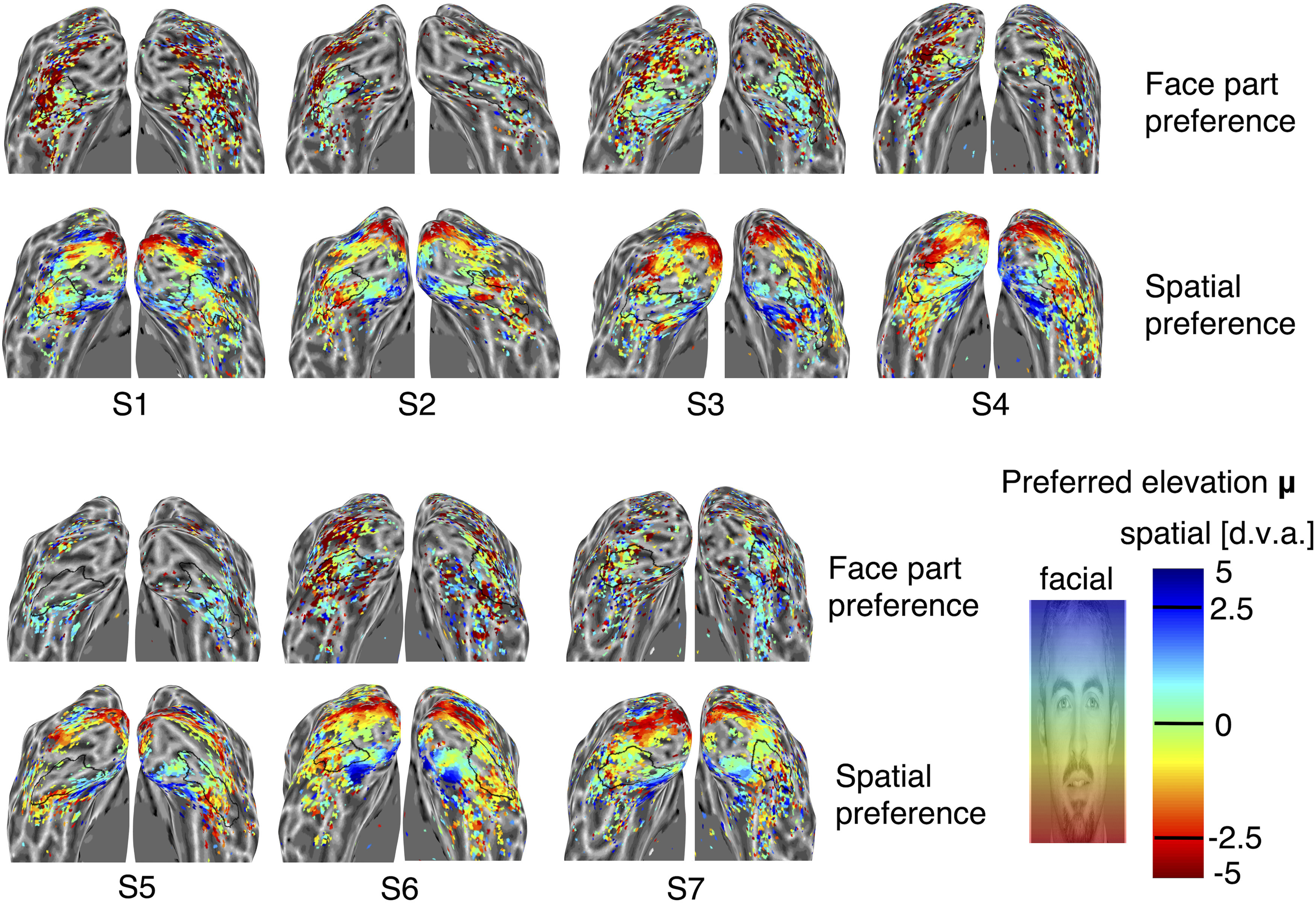
Unsmoothed maps of face-part and spatial preferences for all hemispheres. The color of each vertex indicates the peak preference of face-part and spatial tuning (upper and lower maps, respectively, as indicated by the labels). Colors correspond to the peak location parameter (μ) of the best fitting Gaussian tuning curve model, as indicated by the color bar (the face image shows color-corresponding locations along vertical face space for face preference maps; d.v.a. values show color-corresponding degrees of visual angle relative to fixation along the vertical meridian for spatial preference maps, with cold and hot colors indicating upper and lower visual field locations, respectively). Black outlines demarcate the boundaries of IOG, determined by the FreeSurfer algorithm based on individual anatomy.

Parameter maps were lightly smoothed (3 mm) based on spherical surface distances for visual inspection only (Figs. 2, 3, unsmoothed maps). All statistical analyses were based on unsmoothed data and unique time series, excluding spurious duplications because of the surface projection.

pRFs were estimated based on the averaged time series across the six retinotopic runs, fitting a two-dimensional Gaussian model using SamSrf 6.15 (https://osf.io/mrzqy/) with default settings. The delineation of hV4 followed the anatomical and functional landmarks described in the study by Winawer and Witthoft (2017).

##### Experimental design and statistical analyses.

IOG was defined individually for each hemisphere, using the FreeSurfer parcellation (Destrieux et al., 2010; de Haas et al., 2016). Note that this includes the sulci abutting the gyrus.

The resulting maps of best fitting parameters were thresholded at *R*^2^ > 0.3, which limited the false discovery rate (FDR) in IOG to *p*_FDR_ < 0.05. The FDR was estimated based on the fraction of false alarms (i.e., threshold crossings) in an empirical null distribution of ∼42,000 white matter time courses (Harvey et al., 2015). This allowed us to estimate the upper bound of *p*_FDR_ as the proportion of false alarms in the null distribution divided by the proportion of threshold-crossing time courses in IOG. Note that this is a conservative (upper bound) estimate of *p*_FDR_.

**Figure 4.**
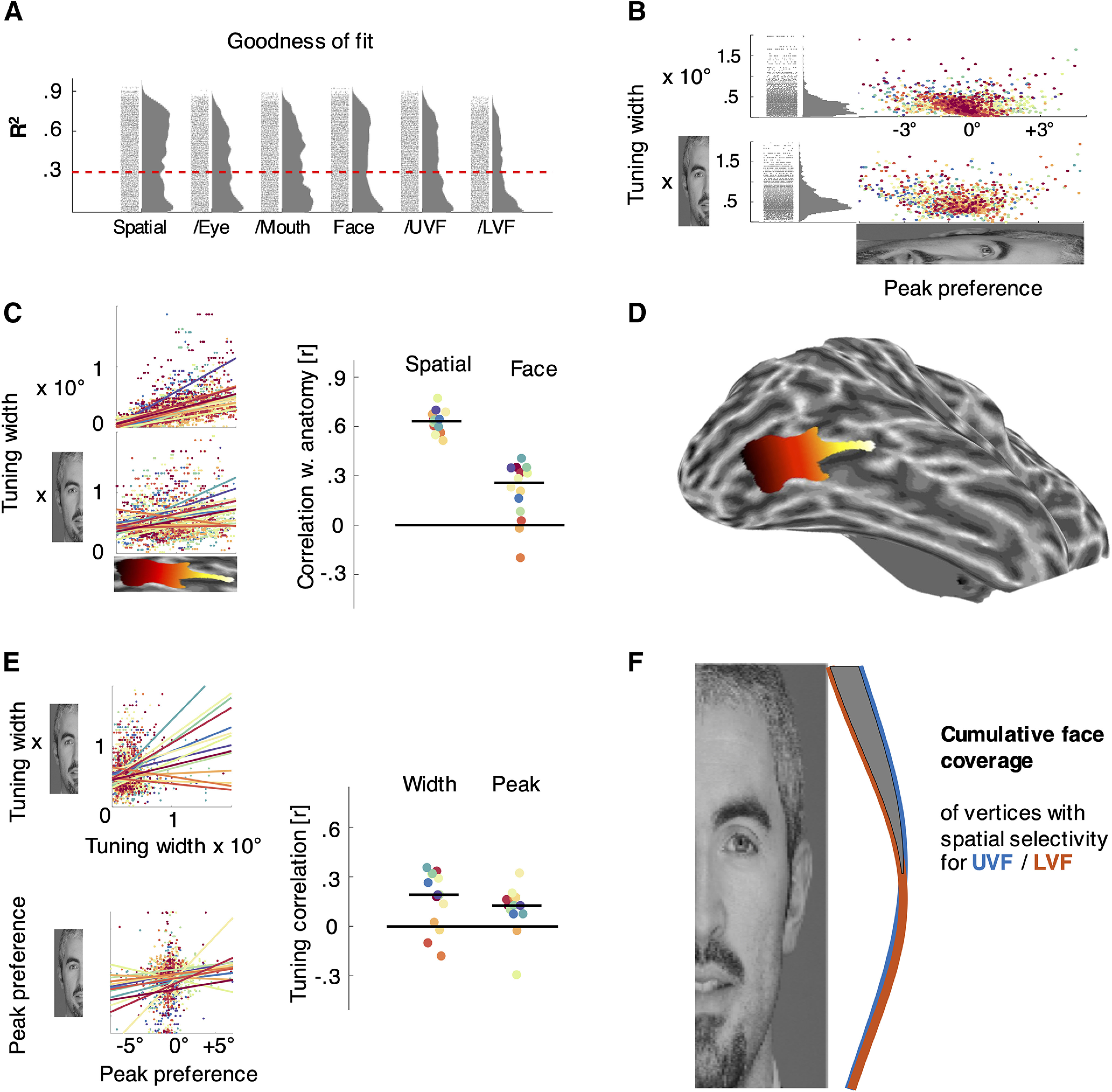
Tuning properties of IOG vertices. ***A***, Goodness of fit for tuning models across vertices. Each histogram shows *R*^2^ values (proportion of variance explained) across pooled IOG vertices from all hemispheres for one type of model. For the main analyses of spatial and face-part tuning, more than half of vertices crossed the chosen threshold of 30% (dashed red line), which corresponds to a false discovery rate of <5% (determined using an empirical null distribution of white matter time courses). Control analysis E further split spatial**-**mapping runs according to stimuli (/eye,/mouth) and face**-**mapping runs according to stimulus location (/UVF,/LVF), yielding significantly worse fits. ***B***, Relationship between width and peak parameters for spatial and face tuning (upper and lower panel, respectively). Each data point corresponds to one vertex, colors indicate individual IOGs. Tuning width significantly increased with the distance of tuning peaks from the horizontal meridian or center of the face. The histograms to the left show the corresponding distributions of tuning widths. ***C***, Correlation between tuning width and anatomy. ***D***, The scatter plots to the left show the posterior–anterior position on the *x*-axis (see the example hemisphere showing the gradient in context). The *y*-axes of the top and bottom scatter plots correspond to the width of spatial and face-part tuning, respectively. Each line indicates the least-squares fit for one IOG. The plot to the right shows corresponding correlation coefficients and marks the median correlation with a black line. ***E***, Correlation of parameters between spatial and face-part tuning. The scatter plots to the left show best fitting spatial and face-part tuning parameters on the *x*-axis and *y*-axis, respectively, plotting the width and peak parameters in the upper and lower panel. Each line indicates the least-squares fit for one IOG. The plot to the right shows corresponding correlation coefficients and marks the median across IOGs with a black line. Note that control analyses confirmed the robustness of these correlations when truncating the range of the data to the 0–1 range. ***F***, Cumulative face coverage of vertices with spatial selectivity for the upper (blue) and lower (orange) visual fields. The shaded region indicates a significant advantage for vertices with selectivity for the UVF. Colors in ***B***, ***C***, and ***E*** indicate data from individual IOGs (14 hemispheres) and are consistent across panels. The ° symbol indicates degrees of visual angle along the vertical meridian, with negative values corresponding to locations in the lower visual field.

To determine whether the width of spatial and face-part tuning followed an anatomical gradient, we manually defined a posterior–anterior axis along the inflated surface of each IOG, taking its individual orientation into account. We then projected the normal of each vertex (in surface space) onto this axis to define its position along the axis and normalized the resulting values to a 0–1 range, with 0 corresponding to the most posterior vertex in a given IOG and 1 to the most anterior one (Fig. 4*D*). This allowed us to correlate the best fitting width parameters (σ) of spatial and face-part tuning of the respective vertices with their position along the posterior–anterior gradient. Hemisphere-wise correlations were Fisher *z*-converted and tested against 0 across the group using one-sample *t* tests with two-sided *p* values.

To determine whether spatial and face-part tuning in IOG are linked, we correlated the best fitting parameters of IOG vertices crossing the threshold across both types of maps (separately for preference (μ) and selectivity (σ) parameters, and individually for each hemisphere). We then Fisher *z*-converted hemisphere-wise correlations and tested whether the mean correlation across the group was different from 0 using one-sample *t* tests with two-sided *p* values.

To test the relationship between width and peak parameters, we computed the correlation between width parameters and absolute distances of peak parameters from the center, separately for spatial and face-part tuning and for each hemisphere.

To test differences in face coverage of vertices preferring the upper and lower visual fields, we pooled data across all hemispheres and considered vertices with very good model fits for both face-part and spatial tuning (*R*^2^ > 50%), as well as narrow spatial tuning (σ < 3 d.v.a.) with peaks at least 1 d.v.a. above or below fixation. We then scaled the face-tuning curves of these vertices to an amplitude of 1 and averaged them separately for vertices peaking in the upper and lower visual fields. This gave us an indication of cumulative coverage along the height of the face. To test differences in the resulting coverage curves for statistical significance, we repeated this procedure 100,000 times, shuffling the labels of vertices peaking in the upper and lower visual fields. We then marked the area between the curves for which the observed distance between them was greater than the distances for any of the shuffles (i.e., permutation *p* < 10^−5^).

To test the robustness of correlations between spatial and face-part tuning and between tuning widths and the posterior–anterior gradient, we conducted several control analyses. Control analysis A consisted of two statistical tests, probing whether these correlations hinged on outliers or extreme values. For the first test, we used nonparametric Spearman correlations instead of Pearson correlations. The second test restricted the data to parameter fits with a selectivity of σ < 1 and peaks inside the mapped stimulus area (0 > μ < 1).

Control analyses B excluded one participant who had very little data above threshold within IOG (S5; compare Figs. 2, 3).

Control analysis C cross-validated the fits of HRF parameters between conditions. The two-pass fitting procedure (see above) was modified to fit separate HRF parameters for spatial and face tuning for each participant. These condition-specific HRFs were then used in the second fitting pass for the respective other condition, ensuring that independent data were used for fitting parameters of the respective HRF and tuning models.

A further control analysis (D) tested whether a bimodal mixture of Gaussians (MoG) model would fit the face-tuning data better than the simple Gaussian model. The MoG model consisted of the sum of two Gaussians with fixed peaks at the eye and mouth locations, respectively. The amplitude and width parameters of either Gaussian were free to vary independently, resulting in one more free parameter compared with the simple model. For each vertex in all IOG maps, we calculated the Bayesian information criterion (BIC; Schwarz, 1978) for the best fit of either model. We then calculated the median difference in BIC values across vertices, to get an indication of the typical evidence in favor of one model over the other (note that higher BIC values indicate worse model performance). We also tested the difference between BIC values for statistical significance using a one-sample *t* test across vertices. Additionally, we manually inspected selected time courses with excellent fits for the MoG model (*R*^2^ > 80%) to check for clear cases of “double peaks” in line with this, but not the simple model.

Finally, control analysis E tested a shift in peak preferences depending on stimulus location or which mapping stimulus was used. Such shifts would be expected if the spatial mapping runs using the eye stimulus yield enhanced responses to upper visual field locations relative to runs using the mouth stimulus; similarly, face-mapping runs at the upper visual field location could yield enhanced responses for the upper face when compared with runs presenting the face parts at the lower visual field location (de Haas et al., 2016). This analysis fit (simple Gaussian) face-tuning models separately for data obtained in runs presenting face parts at the upper and lower central visual fields, and retinotopic tuning models separately for data from runs using eyes or mouths as mapping stimuli. We then tested whether there was an overall shift of preferences by comparing the median height of face-tuning peaks in a given IOG between maps obtained using the upper and lower stimulus locations. Similarly, we compared the median height of spatial tuning peaks between the eye and mouth stimuli. Additionally, we calculated the median goodness of fit for the overall spatial and face-tuning models and for those obtained with separate fits for either location or stimulus. To test pairwise differences in goodness of fit between these conditions for statistical significance, we randomly permuted the respective *R*^2^ distributions 10,000 times and computed the proportion of random differences that was larger than the differences we actually observed.

##### Data availability.

Data and code reproducing the results presented here are freely available at https://osf.io/9gkwx.

## Results

**Figure 1.**
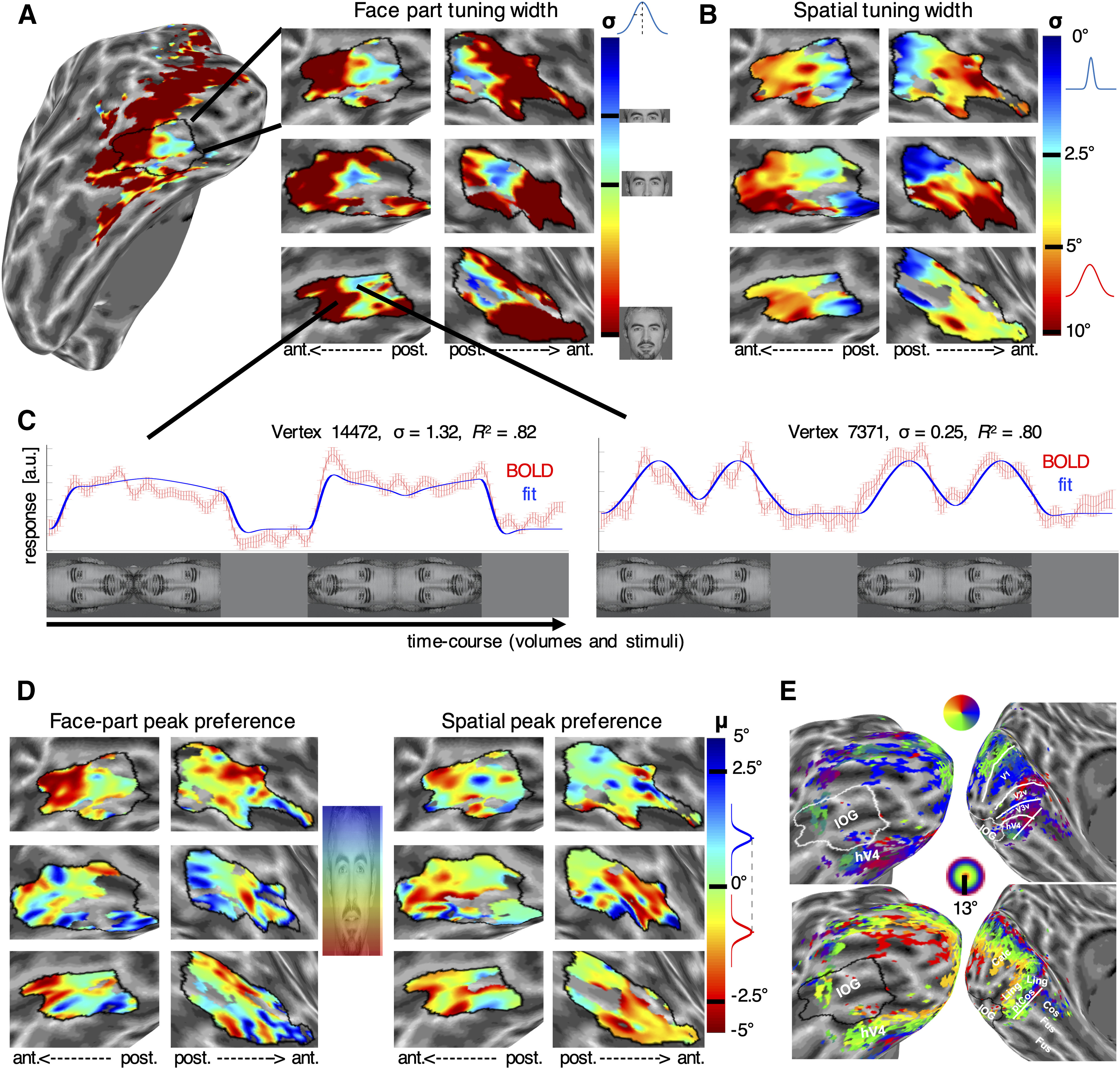
Spatial and face-part tuning in inferior occipital gyrus. ***A***, Posterior–anterior gradient of decreasing face-part selectivity in the IOG of six hemispheres. Map color indicates face-part selectivity according to the best fitting face-tuning model (width parameter σ denotes the SD of a Gaussian tuning curve along vertical face space, as shown next to the color bar). ***B***, Corresponding maps for spatial selectivity, showing a similar gradient (color denotes σ in log scaled degrees visual angle (d.v.a.)) in a log-scaled fashion, as shown by the color bar. ***C***, Example time courses (red) and model predictions (blue) in response to face-part stimuli for two vertices from anterior and posterior IOG (as shown by the connecting lines). Empirical data show the mean amplitude ±1 SEM across runs for each time point (TR = 1 s). The *x*-axis is labeled with the face-part stimuli shown at corresponding TRs. Banding results from overlapping steps in stimulus apertures across volumes. Note that parts from a single face are shown for illustration purposes only. During the experiment, each face part rapidly cycled through facial identities at a frequency of 6 Hz. ***D***, Face-part and spatial elevation preference in IOG. Map color shows the preferred (μ) face-part or spatial location as shown next to the color bar (spatial elevation preference in d.v.a., with negative and positive values corresponding to the lower and upper visual field, respectively, and a logistic modulation of the color bar). ***E***, Position of IOG relative to visual field maps in early visual cortex. IOG is anterior and lateral to hV4. All maps in left and right columns show left and right hemisphere data, respectively, with the anterior (ant.)–posterior (post.) axis oriented as labeled. Black outlines demarcate the boundaries of IOG, determined by the FreeSurfer algorithm based on individual anatomy. Unsmoothed full maps for all hemispheres are shown in Figure 2 (selectivity) and Figure 3 (preference).

For spatial mapping, participants fixated centrally, while an isolated face part slowly traversed up and down the visual field (eyes or mouth, rapidly cycling through different facial identities; Movie 1). For face-part mapping, stimuli were presented at a fixed location (above or below fixation) and sampled from different parts of the face, to slowly traverse a vertical “face-space” (while rapidly cycling through different identities; Fig. 1*C*, Movie 2), and fixation compliance was monitored online using an eyetracker and fed back in a gaze-contingent fashion. Stimulus-directed attention was ensured with a demanding recognition task for a target facial identity.

IOG was defined anatomically on an individual basis, using the FreeSurfer parcellation algorithm (Destrieux et al., 2010). A standard retinotopic mapping experiment in one our participants showed that IOG is anterior and lateral to hV4 (Fig. 1*E*; Winawer and Witthoft, 2017). It was also much less responsive than early visual cortex to our retinotopic mapping stimulus defined by natural images (van Dijk et al., 2016), though this may partly be related to foveal data loss (Fig. 1*E*).

The Gaussian tuning model yielded two parameters of interest for each vertex. The SD of the tuning curve (σ) indicated (inverse) spatial or face-part selectivity (Fig. 1*A*). The peak tuning location (μ) indicated the maximally preferred spatial location or face part (Fig. 1*D*). All maps and statistical calculations were limited to time series with a goodness of fit *R*^2^ > 30% (*p*_FDR_ < 0.05, as determined with a white matter time series null distribution).

Across hemispheres, 56% of IOG vertices crossed this threshold for face-part tuning and 63% for spatial tuning. The median *R*^2^ for spatial and face-part tuning were 43% and 36%, respectively (Fig. 4*A*). A permutation test across all vertices showed that this advantage for spatial-tuning fits was significant (*p* < 0.0001).

Maps of spatial and face-part selectivity showed a clear gradient of decreasing selectivity from posterior to anterior IOG in most hemispheres (Figs. 1*A*,*B*, 2). Figure 1*C* shows time courses (red) and best fitting model predictions (blue) for two example posterior and anterior vertices from a single IOG with excellent model fits. The anterior example (Fig. 1*C*, left-hand side) has a broad width parameter (σ > 1) and shows ramp-like tuning with a preference for the lower end of the face. The posterior example (Fig. 1*C*, right-hand side) shows relatively narrow tuning for the central face region (σ = 0.25; Fig. 1*A*, color bar; note that neural response predictions are convolved with a hemodynamic response function, leading to additional dispersion).

The median correlation between the width of tuning functions and the position of vertices along a manually defined posterior–anterior axis (Fig. 4*D*) was *r* = 0.63 for spatial tuning and *r* = 0.26 for face-part tuning (Fig. 4*C*). Both relationships were statistically significant across hemispheres (*t*_(13)_ = 24.32, *p* < 10^−11^ and *t*_(13)_ = 4.39, *p* < 0.001, respectively; all correlations were calculated separately for each hemisphere, *z* converted, and tested against 0 using one-sample *t* tests with two-sided *p* values across the group). Maps of spatial and face-part selectivity were further significantly correlated with each other (*t*_(13)_ = 3.61, *p* = 0.003; median, *r* = 0.19; Fig. 4*E*).

Note that strong spatial selectivity was widespread across posterior occipital cortex (as expected for early visual areas), while narrow tuning for face parts was concentrated in posterior IOG (Fig. 2).

Parameter maps for peak tuning location (μ) revealed that neuronal populations in IOG clustered according to face-part preferences, but followed a more idiosyncratic organization (Figs. 1*D*, 3). While some maps appeared to follow a gradient along vertical face space, its polarity could be reversed, even across hemispheres of the same participant (Figs. 1*D*, top row). Other maps did not appear to follow a clear gradient or one-to-one face mapping at all. Furthermore, face-tuning peaks concentrated in the central face region, spanning from the eyes to just over the mouth (Fig. 4*B*). Nevertheless, peak preferences in spatial and face space were significantly correlated with each other (*t*_(13)_ = 2.87, *p* = 0.013; median, *r* = 0.13; Fig. 4*E*), and there was a tendency for tuning curves peaking near the horizontal meridian or central face to cluster posteriorly. Vertices with a spatial preference at or near the horizontal meridian appeared highly variable in their face-part preferences, whereas vertices peaking higher or lower in the visual field showed a bias to do_so_in the face as well (Fig. 4*E*). Interestingly, the face-part tuning of many vertices clearly peaked in the lower half of the face (compare Fig. 1*D*, hotter/red colors, 4*B*). This contrasts with face-part tuning in the posterior lateral face patch of macaques, which is dominated almost exclusively by selectivity for the eye region (Issa and DiCarlo, 2012) and is thought to be a homolog of IOG.

To explore the relationship between tuning parameters, we plotted the width of tuning functions against their peak locations (Fig. 4*B*, top, spatial tuning, bottom, face-part tuning). This revealed a strong correlation between the width of tuning functions and the distance of the corresponding peaks from the center of the face or the horizontal meridian (median *r* across hemispheres = 0.51 for both spatial and face-part tuning; *t*_(13)_ = 10.81, *p* < 10^−7^ and *t*_(13)_) = 10.60, *p* < 10^−7^, respectively). This matches the earlier observation of wide tuning curves showing a shallow, ramp-like preference for the upper or lower extremes of the face (Fig. 1*C*). It further mirrors the relationship between eccentricity and receptive field sizes in early visual cortex and may relate to findings of ramp-like tuning for facial properties in the macaque middle face patch (Freiwald et al., 2009).

To further explore the contingency of retinotopic and face-part tuning, we visualized the face coverage of vertices with retinotopic tuning for the upper and lower visual fields. For this analysis, we considered vertices with very good model fits (*R*^2^ > 50%) and relatively narrow spatial tuning (σ < 3 d.v.a.), peaking at least 1 d.v.a. above and below fixation, respectively. We then plotted the cumulative face coverage, separately for vertices with either type of spatial preference. The cumulative coverage of both types of vertices peaked near the center of the face, but slightly higher for vertices with a preference for the UVF. Vertices with a preference for the LVF showed only a small, nonsignificant trend for stronger coverage of the lower face. However, vertices with a preference for the upper visual field showed clearly enhanced coverage of the upper face (*p* < 10^−5^; Fig. 4*F*, shaded area between curves).

We conducted several control analyses. The first aimed to test the robustness of the correlations between face-part and spatial tuning and between the width of tuning functions and the posterior–anterior gradient. Specifically, control analysis A probed whether these relationships hinged on extreme values or outliers by using nonparametric Spearman instead of Pearson correlations. Additionally, it tested whether the relationship between spatial and face-part tuning held when excluding vertices with little selectivity (i.e., tuning curves with σ > 1) and those peaking outside the mapped stimulus area (i.e., tuning curves with μ > 1 or < 0). The relationship between spatial and face-part preference maps (μ) proved robust across both controls (*t*_(13)_ = 3.60, *p* = 0.003 and *t*_(13)_ = 3.50, *p* = 0.004, for Spearman correlations and restricted range, respectively). The same was true for the correlation between spatial and face-part selectivity maps (σ; *t*_(13)_ = 2.46, *p* = 0.03 and *t*_(13)_ = 2.33, *p* = 0.04). Similarly, the correlation between tuning width and the posterior–anterior gradient held in both control analyses for spatial selectivity (*t*_(13)_ = 21.23, *p* < 10^−10^ and *t*_(13)_ = 22.55, *p* < 10^−11^), as well as face-part selectivity (*t*_(13)_ = 4.21, *p* = 0.001 and *t*_(13)_ = 2.40, *p* = 0.03).

Control analyses B excluded one participant with very little data above threshold within IOG (S5; Figs. 2, 3). Again, the correlation between spatial and face-part tuning proved robust for both peak (*t*_(11)_ = 5.80, *p* = 0.0001) and width (*t*_(11)_ = 2.90, *p* = 0.014) parameters. The same was true for the correlation between the posterior–anterior gradient and the width of spatial tuning (*t*_(11)_ = 27.40, *p* < 10^−10^) as well as face-part tuning (*t*_(11)_ = 3.89, *p* = 0.003).

Control analysis C used cross-validated fits of HRF parameters between conditions to guard against overfitting. Here also, the correlation between spatial and face-part preference maps (*t*_(13)_ = 3.15, *p* = 0.008), as well as the one between spatial and face-part selectivity maps (*t*_(13)_ = 3.16, *p* = 0.008), proved robust. The same was true for the correlation between the anatomical posterior–anterior gradient and the width of spatial tuning (*t*_(13)_ = 28.47, *p* < 10–12) as well as face tuning (*t*_(13)_ = 4.24, *p* = 0.001).

Control analysis D allowed for the possibility of multimodal tuning curves. Specifically, we tested whether a bimodal MoG model with fixed peaks at the eye and mouth locations would fit the face-tuning data better than our simple Gaussian model. Given the importance of the eyes and mouth for face perception (Omer et al., 2019), one may expect such tuning, especially if eye and mouth preferring units intermingle at the spatial resolution of fMRI.

Across vertices, the median difference of BIC (Schwarz, 1978) values for the MoG minus the simple Gaussian model was 4.78, providing positive to strong evidence in favor of the simple model. This advantage of the simple model was highly significant across vertices (*t*_(23,807)_ = 34.54, *p* < 10^−10^). Manual inspection of time courses with good fits for the MoG model did not reveal any clear cases of “double peaks” either.

All main analyses used time courses that were averaged across the upper and lower stimulus locations used for face-part mapping and averaged across the eye and mouth stimuli for spatial mapping. However, control analysis E analyzed these four types of runs separately, to test the potential effects of mapping stimuli on spatial tuning or stimulus location on face-part tuning. Given previous evidence for feature–location interactions (de Haas et al., 2016; de Haas and Schwarzkopf, 2018), one may expect, for example, a stronger response in the upper visual field for eye stimuli compared with mouth stimuli and thus an apparent upward shift of retinotopic tuning.

Relative to the main analyses, the goodness of spatial tuning fits was significantly reduced for this separation, for both eye and mouth stimuli (both, *p* < 0.0001; median *R*^2^, 31% and 32%, respectively). Similarly, the goodness of fit for face-tuning models was reduced when they were conducted separately for the upper and lower locations (both, *p* < 0.0001; median *R*^2^, 30% and 22%, respectively). Fits for data obtained in the upper location were significantly better compared with the lower location (*p* < 0.0001). Comparing the best fitting parameters, we found no evidence for a shift of face tuning depending on stimulus location (*t*_(13)_ = 0.33, *p* = 0.75, n.s.). While retinotopic tuning for eye stimuli was lower in the visual field than that obtained for mouth stimuli, this effect was not significant either (*t*_(13)_ = −2.14, *p* = 0.05, n.s.).

## Discussion

We found that the response of vertices in IOG could be well explained by Gaussian tuning curves for retinotopic location and also for the relative position within a face. Parameter maps and quantitative analyses revealed a common posterior–anterior gradient for retinotopic and face-part selectivity. Retinotopic and face-part tuning parameters were correlated with each other. The cumulative face tuning of vertices with a narrow retinotopic preference for the upper versus lower visual field was significantly stronger in the upper face than that of vertices with a narrow retinotopic preference for the lower visual field.

Receptive fields have long been known to increase in size and in scatter moving anteriorly, implying less spatial precision; however, the finding that feature selectivity decreases in tandem is novel. These findings support the hypothesis that face-part preferences in IOG are linked to retinotopic tuning (Henriksson et al., 2015; van den Hurk et al., 2015; de Haas et al., 2016) and may provide a neural basis for recently discovered feature–location interactions in face perception (de Haas et al., 2016; de Haas and Schwarzkopf, 2018). As such, they are in line with the more general proposal that the functional organization of the ventral stream is shaped by an interplay of retinotopic maps with typical gaze behavior (Arcaro et al., 2019; Kaiser et al., 2019; Livingstone et al., 2019). The canonical axis of functional organization in IOG seems to be a posterior–anterior gradient of decreasing spatial and face-part selectivity (Figs. 1*A*,*B*, 2, 4*C*). The functional organization of spatial and face-part preferences (i.e., tuning peaks) was more idiosyncratic, but nevertheless correlated across face-part and spatial domains. This may reflect a greater degree of developmental plasticity for peak preferences, possibly linked to individual differences in gaze behavior. Note, however, that previous retinotopy studies indicate that IOG may overlap with two (shape-preferring) contralateral hemifield maps (the dorsal and ventral putative human poster inferior temporal cortex maps; Kolster et al., 2010). The concentration of face-tuning peaks in the central face region, spanning from the eyes to just over the mouth, matches the distribution of typical and individually optimal landing points for face-directed saccades (Peterson and Eckstein, 2012, 2013). It is noteworthy that vertices with a spatial preference at or close to the horizontal meridian displayed a range of face-part preferences covering the whole inner face region, whereas vertices peaking higher or lower in the visual field had a stronger bias for matching face-part preferences (Fig. 4*E*). This appears in line with typical gaze behavior, which foveates various inner features but still results in matching feature biases for upper and lower visual field locations (de Haas et al., 2016).

Interestingly, the face-part tuning of many vertices in IOG peaked for the lower half of the face (Fig. 1*D*). This is in contrast to earlier findings in the posterior lateral face patch of macaque monkeys, which is heavily dominated by a preference for the contralateral eye region and thought to be the homolog of IOG (Issa and DiCarlo, 2012). This divergence may point to the special importance of the mouth region in human communication (Rennig and Beauchamp, 2018; Rennig et al., 2020). However, we cannot exclude the possibility that other differences in methods and design contributed to this difference. For instance, most eye-preferring units in the study by Issa and DiCarlo (2012) had a peak preference for the contralateral upper visual field at eccentricities beyond that of our upper visual field location, which could theoretically lead to an under-representation of eye-preferring units for our study.

Our results suggest that narrow face-part tuning is a specialty of IOG. The underlying gradient of spatial selectivity appears part of the wider (and well known) retinotopic organization of occipital cortex, with increasing receptive field sizes from posterior to anterior (Fig. 2). Narrow face-part tuning seems much more concentrated to posterior IOG and its vicinity (Fig. 2). Note, however, that our design was optimized for IOG as a region of interest, with a posterior coil arrangement and a slice package with partial coverage (Material and Methods). It is possible that other face patches, for instance in the posterior and anterior fusiform gyrus, are organized in a similar fashion [Fig. 2, right hemisphere of S7 (which appears to show multiple reversals of face-part selectivity along ventral temporal cortex)]. Future studies should extend our approach to a detailed study of all face patches. Developmental studies may be able to disentangle whether retinotopic tuning gradients and face tuning co-occur or one precedes the other (Arcaro et al., 2017; Gomez et al., 2018).

An important implication of our finding is that “part-based” and “holistic” processing appear poles of a continuum. Neural populations in IOG cover a whole range of face-part selectivity and are organized along a corresponding gradient. Therefore, it seems unlikely that the computational role of this area is well captured by a blanket label of part- or feature-based processing. This contrasts with influential “box-and-arrow” models of neural face processing (Haxby et al., 2000; Duchaine and Yovel, 2015) and should inform the design and interpretation of neural network models (Grill-Spector et al., 2018).

There are several limitations to the current study. Most importantly, blood oxygenation level-dependent (BOLD) signals in any given voxel reflect responses from tens of thousands of neurons, with potentially diverse tuning properties. While fMRI provides an excellent tool for discovering broad gradients of neural tuning or cortical patches with common preferences, it can miss or even disguise underlying local heterogeneity (Sato et al., 2013). This problem may be alleviated somewhat by the increased signal-to-noise ratio and spatial resolution of higher field strengths, but ultimately is determined by the point-spread function of the BOLD signal and underscores the need for intracranial recordings.

The simple Gaussian model of face-part tuning we tested here provided good fits to the data and outperformed a bimodal mixture of Gaussians model. However, our study used a stimulus that continuously traveled the visual field or “face space.” Given the slow nature of the BOLD response, such a design likely enhances the power to detect smooth unimodal tuning at the expense of sensitivity for tuning curves with multiple or more narrow spikes. Moreover, recent results show that such orderly designs increase the susceptibility of spatial tuning fits to design choices, such as cycle duration (Infanti and Schwarzkopf, 2020). Future studies could present face samples in a random order (Kay et al., 2015) and explore tuning properties using model-free reverse correlation approaches (Lee et al., 2013).

Splitting retinotopic and face-mapping runs according to stimulus (eye/mouth) and location (UVF/LVF) revealed no significant shifts of overall tuning. This is in contrast to what may be expected if face parts at typical locations evoke stronger responses, as suggested by the feature–location interactions in human recognition performance (de Haas et al., 2016; de Haas and Schwarzkopf, 2018). For instance, if eyes evoke stronger responses when presented in the upper visual field, the apparent spatial tuning of vertices may shift upward when using eye stimuli rather than mouth stimuli for mapping. However, we observed no such effect in our data and even a trend in the opposite direction. This appears to be at odds with the clear evidence for increased coverage of the upper face in vertices with selectivity for the upper visual field (Fig. 4*F*). Split analyses also resulted in significantly worse model fits, further limiting their interpretability. Nevertheless, this unclear finding demonstrates that the neural basis of feature–location interactions is not yet settled, although tuning correlations in IOG appear to be a likely candidate.

Face recognition performance generally varies as a function of visual field location (Peterson and Eckstein, 2012), and this can be subject to idiosyncratic biases (Afraz et al., 2010; Peterson and Eckstein, 2013; Peterson et al., 2016). Here, we only tested two locations for face-part mapping and found significantly worse fits for the lower visual field location, which also had higher eccentricity (Fig. 4*A*). Future studies could probe this anisotropy more systematically and correlate it with corresponding individual biases in face perception and fixations.

Our design presented face photographs at a fast pace known to enhance responses in face-preferring areas (Stigliani et al., 2015). However, previous studies in macaque successfully used cartoon stimuli to probe face tuning (Freiwald et al., 2009), which allow greater control. For instance, the eye region in natural images is usually less variable than the mouth region and thus easier to align (compare Movie 2), which may have unknown effects. More generally, future studies could use artificial stimuli to test the role of low-level features, such as contrast polarity (Ohayon et al., 2012).

Our spatial tuning results are in line with earlier findings (Kay et al., 2015) showing coarse population-receptive fields in IOG, as well as a positive relationship between their size and eccentricity (Fig. 4*B*). However, this previous study also showed that the spatial tuning of individual vertices becomes coarser when the participant attends the stimulus. In our experiment, participants completed a demanding stimulus-directed task, potentially widening tuning curves. Importantly, the potential task dependence of face-part tuning has yet to be tested.

In conclusion, vertex-wise tuning models revealed that human IOG is organized into correlated gradients of spatial and face-part selectivity. This link may be shaped by typical face-directed gaze behavior and provide the neural basis of feature–location interactions in perception. Feature gradients following retinotopic tuning may be a general organization principle within the ventral stream.
